# Stand up for the New and the True

**Published:** 1887-12

**Authors:** E. D. Babbitt

**Affiliations:** New York College of Magnetics, 39 West 27th St.


### STAND UP FOR THE NEW AND THE TRUE.

BY E. D. BABBITT, M. D.

There seems to be a wonderful perversity among many minds which leads them to cling to the old and the false. Prayer books will copy the prayers that were offered one or two thousand years ago in the dark ages, in preference to those of the more enlightened present. Medical men cling with terrible tenacity to such dangerous and health destroying drugs as mercury, antimony, opium and the like, and such fierce methods as burning, leaching and carving, when all around them are such glorious upbuilding influences as sunlight, air, water and the magnetism of highly charged human beings. In the ignorant, superstitious past, people concluded there was good luck in a senseless horse-shoe, and so you will see old rusty horse-shoes kept in hallways as a sacred protection, and horse-shoe toys and trinkets kept for sale in shops. One of the most senseless of all superstitions is the supposition that Friday is an unlucky day, and many people that should know better, fear to commence a new business on that day, and some intelligent people really believe that there is a fatality in the number thirteen, as though such a dead thing as a number, could wreak some kind of vengeance upon them. But one of the most disastrous things is to run forever in old ruts, to follow constantly in beaten paths because our forefathers did, or because our books and teachers have commanded us to do so. This could be illustrated by a thousand examples, but I will content myself with one or two. I remember the case of a Dr. Dennis, a first-class dentist of Cincinnati. This gentleman discovered a method of covering up the exposed nerves of teeth with a plastic substance. In this way he prevented a vast amount of suffering, and preserved living nerves instead of dead putrifying ones, on the old plan His method was tested in a multitude of cases with constant success. Did all of the dentists adopt his plan as they should have done? No. A few of the larger souls adopted it with great success, but the mass of them ignored it, and even the College of Dentistry of that city, was afraid of a new thing, so that the doctor, disheartened at such backwardness, ceased trying to enlighten his fellow dentists. What is a college good for, if it cannot stand in the vanguard of progress and lead the profession upward?

Let me mention one more case. Dr. Wm. E. Dunn, one of the old and skilled dentists of New York City, long ago discovered that rubber plates for teeth were very apt to contain mercury as well as sulphur and other heating elements ; that celluloid was also thermal and inflaming to the mouth, and that the galvanic action of metal plates was highly detrimental to the health. He devised a kind of a porcelain substance, composed mainly of feldspar and silex, to be used in their stead. The change thus produced was in many cases startling. Lives were saved, deafness was, in numerous cases cured, terribly inflamed and salivated mouths were healed almost immediately, and systems shattered by dyspepsia and paralysis were saved by the new cooling influence. A moment's thought would show that heating elements should never be used so near the brain as the mouth. Peter Cooper's health was almost revolutionized by the new process ; Mrs. C. S. Lozier, M. D., speaks of the "incalculable benefit" of the new method in her own case ; Dr. J. R. Buchanan calls it "the greatest improvement ever made in dentistry" and many other physicians speak in glowing terms of it. But how have dentists themselves received it? Only a few have been sufficiently enterprising to adopt it ; members of the New York College of Dentistry, though privately aware of the excellencies of the new system, make no public explanation of it to their students, and the new work in three large volumes called "The American System of Dentistry," makes no mention of it. Thus do the timid old fogies ever keep the people in the background, and allow them to continue to suffer and agonize without lifting a hand to help them.

But do not imagine, dear reader, that I am advocating the importance of all new things. Human minds are prone to be narrow or one-sided or capable of grasping only one idea at a time. Some people learn that fine effects can be produced by the mind cure, and so they conclude that everything can be done by the mind, and, running off in a tangent, neglect a whole universe of upbuilding forces outside of themselves. Some consider the water cure all that is needed, some the air cure, some exercise, some advocate a single color of the sunlight, while but few are large enough to advocate the whole spectrum of colors and the whole range of forces. As magazine articles should be short, I must omit many things which might be profitably considered. Before closing, however, I wish to mention briefly the theory of Dr. Jaeger of Germany, whose form of monomania seems to begin and end with *wool*. The whole world of cotton that we receive so abundantly and cheaply, is evidently, according to his theory, one of nature's mistakes. He advocates only woolen garments next to the skin, woolen stockings, woolen sheets to sleep in, and,

I believe, woolen shoes. Wool is best, he thinks, because it shuts in the vital radiations. But he seems to forget that it also shuts in the poisonous emanations of carbonic acid for the skin to re-absorb, and shuts out the electricity of the air, which is important for the body. Wool is good, also, because it absorbs moisture, he says, but merino and any other cloth that has a nap, will also absorb moisture, and will not be forever shrinking, as is the case with pure wool. But shall I say never use woolen garments next to the skin ? No, for that would be fanaticism in another direction. While I deem cotton or merino best for most people, next to the skin, and especially cotton for sheets, many elderly people and many thin, bloodless people may find woolen undergarments best. The irritating effect of wool, too, may sometimes be animating to the skin, though it is intolerable to many fair and thin skinned people. I have spoken of this heresy the more freely because it is spreading in this country, and people are being taught in various directions, that wool is the only thing fit to wear.

*New York College of Magnetics*, 39 *West* 27*th St.*

